# Book Reviews

**Published:** 1818-07

**Authors:** 


### CRITICAL ANALYSIS
### *OF RECENT PUBLICATIONS,*
IN THE
### DIFFERENT BRANCHES OF PHYSIC, SURGERY, &c.

*Surgical Observations; being a Quarterly Report of Cases in Surgery; treated in the Middlesex Hospital, in the Cancer Establishment, and in private Practice. Embracing an Account of the Anatomical and Pathological Researches in the School of Windmill Street.* By CHARLES BELL. Vol. II. Longman and Co.

MR. BELL we believe to be a good man, and, if not a good writer, is at least a prolific one. We have before us the fifth part of a work, which, from a charitable wish to preserve the reputation of the author, we would rather had not seen the light. There is perhaps, too, something of personal feeling in this; for we should have been spared the pains, imposed on us by the duties of our office, of wading through heaps of nonsense without the consolation of acquiring a single idea on our way. The first of these Reports is entitled, "the Substance of Clinical Remarks delivered in the Hospital, in which the formidable nature of the Syphilitic Ulcer is described; to which is now added, some Observations on late Changes in the Practice of the British Army." "Just about the time (says Mr. Bell) when a *safe rule of practice* in the treatment of the venereal disease had been established among judicious surgeons, when the experiments and theories of Mr. Hunter had served to give a more correct idea of syphilis, and a simple mode of practice, certain opinions came into fashion, *which have increased in extravagance, and have been attended with the very worst consequences.*" Mark, reader—a safe rule of practice! If you know any thing of constitutions broken up, noses dropping off, fauces and other parts eroded by phagædenic ulcerations, exfoliations of the palate, nocturnal pains, and all the miseries of this disease subsequent to the regular use of mercury, you will be racked with impatience until you learn what this safe practice can be. Mercury, it appears, is the panacea, which it is the sum and substance of this report to eulogise; and this is done with as much arrogance, on the part of the author, as could well be evinced, and as large a portion of sneering insinuation against his adversaries as decency would permit. Without pledging ourselves to the truth of the assertions again prevalent,—that the venereal disease is absolutely curable without mercury, although evidently proved to be so in many well-authenticated cases, we feel ourselves bound to condemn the manner in which the question has been handled by Mr. Bell. He would have rendered a more acceptable service to the profession, if, instead of dealing so largely in general assertion, he had either proved the falsehood of the opposing statements, or refuted the conclusions deduced from them. Nothing of this is attempted ; but he talks with the most perfect conceit of the extravagance of all opinions on the subject but his own, and assigns to them consequences without the shadow of a proof in his justification.

After the preceding quotation, which is followed up by a page full of sublime sentiments about the necessity of attending to the health and welfare of soldiers, with a strong but unnecessary assurance, that " we, of the civil department, do hold the interests of our soldiers as near our hearts," the author sets to work, and begins by telling us, that an opinion is very current among our young practitioners, (he might probably have added among apple-women also,) " that it (the disease) is dying out like the old stock of apples!" We do not think it necessary to follow Mr. Bell in the work of refuting that which nobody in his senses can believe ; but it may be proper to mention in this place, that, in every age, as far as the records of physic will carry us, the venereal disease has been uniformly cured by some practitioners without mercury. Fallopius, whose authority is undoubted, tells us, at the close of a long practice, that, in *one case*, he was obliged to use mercury, but that the radical cure of the disease was to be found in guaiacum.

If the opinion referred to by Mr. Bell has obtained currency among the weaker part of our brethren, it must have arisen from the body of evidence adduced by the anti-mercurialists to prove that syphilis is capable of being cured without mercury. The answer then is obvious, since it appears, that, in the times of the older surgeons, the same facts were known; and, as the disease was then also curable without mercury, and frequently aggravated and rendered incurable by it, we have no reason for suspecting a shade of difference, either in the nature or the severity of its symptoms.

Among other specimens of curious reasoning, the following stares us in the face.

" Now the misfortune is, that ***hundreds*** and ***thousands*** of cases, brought from the practice of the army in support of opinions, must necessarily produce a sort of revolution in the mind. A man cannot help feeling staggered, in his most established opinions, by a body of evidence apparently so overwhelming ; but, when we return to the contemplation of two or three well-marked cases, some obstinate facts, standing like land-marks to guide us, we return to the dictates of sober sense, and to the long experience on which *our safe practice* has been grounded."

This recurrence of Mr. Bell to his safe practice, bears too near a resemblance to modern quackery;—but, not to digress, it seems most unfortunately admitted by him, that *hundreds and thousands of cases* are adduced by the army surgeons, while "we, of the civil department," oppose to them two or three. Surely it cannot be a source of wonder on which side of the scale the evidence will preponderate. The two or three, for we reason on his own facts, will be but as exceptions to the general rule; and it may still, as before observed, be a question, whether their obstinacy and intractable nature may not have arisen from bad treatment, or from peculiarity of constitution. This we may remark, without any pledge to adopt either side of the question, but simply as fair and legitimate conclusions from the premises, since it is impossible to prove that the cases were absolutely incurable without mercury. The probability is, that "*in medio stat veritas.*"

We fully agree with the author in the necessity of guarding his pupils against the hasty formation of opinions, but we think him not warranted in anticipating the disappointment prepared for themselves by the advocates of the contrary practice, and the misery of which they are laying the foundations.

Mr. Bell conceives the army surgeons have either not seen, or have not chosen to describe, the venereal ulcer, and supplies us with extracts from his note-book as examples of the real disease. These we quote for the purpose of putting them in contrast with the descriptions of Mr. Rose's cases, and shall leave the reader to form his own conclusions. We confess we see nothing in them peculiarly characteristic of syphilis, which those of Mr. Rose do not possess; and, had they occurred in our own practice, we should have at least *attempted* the treatment without mercury,—and it will be observed, that, in most of the patients, no other remedies were previously tried, or at least none referred to. The mere circumstance of mercury having effected the cure, proves nothing, while we admit that a variety of diseases are curable by the same remedy. Nor is it clear that mercury did effect the cure, since it is well known that rest, attention to diet, and temperance, are of themselves capable of effecting considerable amendment in similar cases. This the author admits by a quotation from Mr. Lagneau, who noticed, that, "when patients, who were received into an hospital, have had to wait the period of entering on a course of mercury, these symptoms have sometimes disappeared by rest, and the use of tisans."

Mr. Bell's examples of genuine syphilis are as follows:—

"*Venereal Ulcer on the Penis.*

"*First Report.*—The penis is very much mutilated in consequence of former chancres; there is a sore seated on the ruins of the old one; it extends from the glands to the scrotum, along the lower part of the penis, and has destroyed the urethra. This patient has taken no mercury.

"*Second Report.*—The ulcer is making progress, notwithstanding that I have put him on a course of mercury.

"*Third Report.*—It is worse; for, though stationary below, and perhaps less foul, it is eating deep by the side of the glans. Is this sore truly venereal, or is it phagædenic? Is it to be cured by pushing the mercurial course, or by a sudden and entire change of measures? I have preferred the first, because the sore is ragged on its edges, and irregular in the bottom; because the secretion within the sore is of a greenish yellow, in some places black and slimy, and fixed to the bottom of the sore (like the slime which is attached to the bottom of running water), and the granulations project through the foul bottom of the sore; because the granulations have on their points small specks of extravasated blood, which on the morrow fade and waste away. The mercury being pushed to salivation, the sore became clean, and in due time healed.

2*d Example.* "A gentleman received the infection at Malta, and travelled across Italy and France without taking mercury. This is the transcript of the note descriptive of his sore: 'There is in the groin an ulcer of the size of my hand; the longest diameter across the upper part of the thigh. It is deep, and very irregular at bottom. There stand up from the bottom, red portions of the size of the uvula, and betwixt these there are chops and chasms of tremendous depth, considering the place of the artery. The edge of the sore is irregular, angular, and ragged; towards the ilium it is of a dark red, the skin thin and undermined. Towards the pubes the edge is semicircular and abrupt, covered with a foul viscid secretion, through which granulations appear obscurely. The outer edge of this margin of the sore is covered with a crust of dark red secretion, which is concreted, and is like dried blood. The progress of the ulceration is by the fading of the granulations. At night you see a spot of extravasated blood, and in the morning, the prominence of that granulation is dull in colour and shrunk, and next day it has disappeared altogether.

"As soon as the mercury darkened the gums, we saw no further progress of the sore, nor wasting of the granulations. The surrounding inflammation disappeared; the edge became clean and suppurated kindly, and day after day contracted, and finally closed.

"3d. The ulcer which has destroyed the septum nasi, has made progress upon the upper lip. The nose is entirely excavated, and the integuments are much swollen. The lip and the nose are separated. The lip is separated from the alveoli, and the nose and mouth communicate before the teeth. The upper lip is very tumid and inflamed; it exhibits a broad foul ulcer where it is separated from the nose, and a viscid dirty yellow slime covers it. There is a dark line, as if formed by concreted bloody matter on the very edge, which, if plucked off or loosened by poultice, would exhibit a raw sharp ragged edge. The hideous appearance, and her fears, present a picture of misery. This has been a woman remarkable on the town; but she affirms, that for years she has not been in danger of infection. I say, notwithstanding, this is venereal. A fortnight after this a mercurial course was begun, after soothing applications, and all those lesser remedies which are called attention to the general health, had proved to be unequal to the cure. Under mercury the sores healed rapidly, but the woman's face was sadly disfigured.

"4th. The ulcer is narrow, deep, and foul, but the depth is more relative to the edge of the sore than to the surface of the cheek; for the edge is elevated into a ridge, which is surmounted with a red line, sharp, and minutely notched. The centre of the sore is lined with a yellowish green matter that cannot be wiped out. The ulcer is extending down the cheek, while, by the aid of escharotics, there is a disposition to close in the upper part. The direction in which the ulcer is to extend, is declared by a minute speck of a bluish colour on the edge of the sore.

"This ulcer, after resisting a great variety of dressings, changed its character, as soon as the mercury made the mouth sore; the elevated ridge subsided, the sharp edge was rounded, the foul secretion could be wiped off the inner surface; granulations appeared; and the skin was drawn in and healed.

"5th. When I look into this man's throat, I find the soft palate in a great part destroyed, and the edge is left irregular, and covered with a snotty viscid secretion, which cannot be washed off, and yet appears as if it might. Around the edge of the ulcer, and spreading on the roof of the mouth, there is a blush of dark inflammation, without tumefaction, in strong contrast of colour with the foulness within. The ulcer is excavated, irregular, and lined with an ash-coloured slough; it appears as if the substance were dug out, leaving a determined edge.—*Venereal Ward, 7th Aug.**

---

"* About this time a singular case occurred:—A man, about forty-five years of age, was sent up to the Venereal Ward from the Physicians' Ward, where he had been considered as rheumatic. It was a complicated case; for, besides foulness of skin, and nodes and pains in the bones, he had a deep ulcer in the throat. Soon after he was brought up, he became paralytic, and, three days

"The mercury has affected the gums; the sore is already clean and diminished in extent.—Aug. 17.

"What is meant by saying that the disease has lost its violence, I am at a loss to conceive, unless it is, that we become day by day more capable of throwing off the unpleasant impressions which continue to haunt the younger imagination. Is not the following description like the picture of the disease in the sixteenth century? I do not vouch for this being the pure effects of syphilis, nor dare to say how much belongs to improper treatment of the disease.

"6th. The nose has been destroyed by ulceration, and the ulcer is still active on the cheek. The lips are separated from the alveoli; they are enormously swollen, and hang loose. Within the cavities of the face, the bones are carious, and there flows from them a very fœtid discharge, excoriating. The eyelids and eyebrows have lost their hair. The margin of the eyelids are tumid and ulcerated; the hair of the head has fallen off, and a garland of inflamed tubercles encircles the forehead. The cornea is dull; the hearing also is much affected. This gentleman still clings to existence; and hopes yet to enjoy life, when he shall have retired to his estates in the pleasantest county of England."

These are the descriptions by which Mr. Bell hopes to establish for ever the characteristics of syphilis, taken too from "my note-book," and intended as an instructive lesson to the army surgeons, who are carried away, as he thinks, by a destructive heresy. Mr. Rose's cases are severally subjoined.

"I.—*Sores which were not followed by any Constitutional Affection.*

"*Case* 1.—A sore of the size of a split pea, where the prepuce joins the corona glandis. The sore was circular, without granulations, with matter adhering to its surface, and with a hard and thickened margin somewhat elevated. He had a similar sore on the outer edge of the prepuce. The sores had been present a fortnight, and came five or six days after suspicious connexion."

"*Case* 2.—A sore as large as a sixpence on the internal prepuce, which had destroyed the margin of the corona glandis. The surface was sloughy; the sore was not very deep, but was attended with a very great degree of induration. It came eight days before his admission, and two days after suspicious connexion."

"*Case* 4.—A deep foul sore close to the corona glandis, with considerable hardness and thickening of its base and margin: had

---

after, died apoplectic. On the examination of his body, the ulcer in the throat was seen to communicate through the basilar process of the occipital bone, with an ulcer of the medulla oblongata; and this ulcer having made progress upwards, had opened the basilar artery."

been present a month, and came about a week after suspicious connexion. It had occasioned a bubo in his right groin, which had suppurated and burst. He was just returned from a detached duty, and from the acrid discharge of the sore, the prepuce was inflamed and excoriated."

"*Case* 5.—The same man was re-admitted (a month after) with a deep ulcer of the corona glandis of the size of a silver penny, and a smaller one immediately bordering on it. A portion of the glans was destroyed; the sores were highly inflamed, with a dark slough in their centres, and with hard and thickened margins. The whole prepuce was thickened and inflamed, and several small ulcers had formed from the acrid discharge. The sores had been present three weeks, and came a week after suspicious connexion."

"*Case* 6.—Joshua Reeves was admitted on the 24th of January, 1816, with a foul irritable sore, having a thickened margin, where the internal prepuce joins the upper part of the corona glandis, to which it had contracted some preternatural adhesions. The sore was of eight days' standing, and came a week after suspicious connexion."

"*Case* 7.—A sore which had eroded underneath the frænum, and had the common appearances of a chancre in that situation. There was considerable induration of the frænum, and margins of the ulcer. He said it had been present only a day or two, and arose from a suspicious connexion a week before."

"*Case* 8.—Two sores on the internal surface of the prepuce, one on each side of the frænum. The sores were circular, and each about two-tenths of an inch in diameter. They had a dark-coloured matter adhering to their surface, were attended with considerable loss of substance, and showed no disposition to granulate. Their edges were irregular, and there was a very considerable induration of their margins and base. The prepuce was inflamed and tense. There was an incipient bubo in his left groin. He observed the sores a few days previous to his admission, and had been exposed to the risk of infection at Portsmouth about a week before. In a week the sores had gradually spread, and complete phymosis had come on."

"*Case* 9.—Six or eight deep irritable sores on his internal prepuce. Their surface was covered with a dark-coloured slough; they had thickened and highly inflamed margins, and discharged a very acrid ichor. He complained of headach and thirst; had a quick pulse and other febrile symptoms. There was a chain of enlarged glands in each groin, which he said was of long standing."

"*Case* 10.—Had partial phymosis: his prepuce swollen and much inflamed, with a most acrid and copious discharge from beneath, excoriating the neighbouring parts. The whole margin of the corona and the upper surface of the glans were covered with small white vesications. Disease commenced three days before."

"*Case* 11.—A chancre, which came ten days after suspicious connexion, and was healed with a great deal of hardness, by means of topical applications, on the 24th of November. On the 1st of that month, a gland inflamed in his right groin which suppurated rapidly, and, as it occasioned a great deal of pain, it was opened with the lancet on the 13th of November."

"II.—*Sores which were followed by Constitutional Symptoms.*

1.—*Papular Eruptions.*

"*Case* 12.—A large but not very deep sore on the edge of the inner membrane of the prepuce. It had no disposition to granulate, and he had an open bubo in each groin. The sore had, from his account, been present a fortnight, and the buboes rather more than a week. The former was healed on the 14th of June; and, on the 24th of that month, after feeling chilly and feverish for two or three days, an eruption of inflamed papulæ, like common lichen, appeared over his body, and about his forehead and neck. On the 28th, the febrile symptoms had disappeared; but the eruption was very thick, and his left eye was inflamed. The inflammation did not appear to have extended to the internal tunics."

"*Case* 13.—An indolent sore close to the edge of the frænum, which had been present nearly two months. It was healed on the 25th of June, with some hardness and thickening, and he was dismissed. Aug. 20; he returned, covered with an eruption of lichen. This he said appeared about a week before, at which time he had severe pains in his thighs and legs. When admitted, the eruption was general over the face and body, and very thick about the arms and legs. The papulæ were small, and here and there filled with an opaque lymph, or puriform fluid; some were beginning to go off in scurf."

"*Case* 14.—A sore at the extremity of the prepuce, having thickened edges and some induration. He had also a bubo on his left groin."

"*Case* 15.—A superficial ulcer of some days' standing, on the outer skin of the prepuce, near its extremity. The glans could not be denuded, in consequence of the contracted state of the prepuce. He had been exposed to the risk of infection a short time before the sore appeared. It healed in three weeks; but, the phymosis proving troublesome, the contracted ring at the end of the prepuce was cut off, and the inner membrane slit open.

"*Case* 16.—Whilst in hospital with pneumonia, was found to be affected with phymosis, attended with much dark-coloured discharge from under the prepuce. The sore could not be brought into view, but he said it was situated near the frænum; that it had been present nearly a month, and came four days after suspicious connexion. He had an open bubo of a fortnight's standing in each groin. The phymosis was so far reduced on the 23d of December, that a deep foul sore was brought into view on the left side of the frænum, of the size of a silver penny, with hard and irregular edges."

"*Case* 17.—21st of February, 1816; attacked two days before with pains in his shoulders, but without any distinct febrile symptoms: an eruption of lichen over every part of his body. The papulæ were large, thickly, but equally diffused, and many of them filled with yellowish lymph. He had also a dark-coloured inflammation of the integuments covering each shin, a little above the ankles. He had a sore on the internal prepuce in Nov. 1815, which was cured in about a month, by a lotion without mercury. This sore appeared a week after suspicious connexion, and left thickening of the cicatrix, and depression from loss of substance."

"*Case* 18.—29th of June, 1816; had occasional pains in his shin-bones and knees, increased when he was warm in bed, but not so severe as to prevent sleep: a very copious eruption of lichen in large papulæ over every part of his body, and inflammation of both irises. There was a deep zone of vessels round the cornea; the pupils were irregular; and large drops of lymph on the margin of each pupil. The pains had been present a month, the eruption a week, and the inflammation of his eyes only three days since: had a similar eruption, though not nearly so copious, about three months before."

"*Case* 19.—Ardor urinæ, purulent discharge from the vagina, and two or three superficial sores, with slightly-thickened edges, on the inner surface of the vestibulum. Nov. 9; she was covered over the face, scalp, and every part of the body and limbs with an eruption of lichen, so minute and so thick that not a particle of sound skin could be discovered; particularly about her forehead, which appeared puffed and swelled. Her tonsils were a little enlarged, but she had no pain in swallowing. The eruption began on the 7th: she had been feverish for a day or two with frequent rigors, headach, pain in her limbs, thirst, loss of appetite and restlessness. The symptoms were still present; her tongue was white, and her pulse about 120. The discharge from the vagina continued, but the sores were healed."

"2. *Constitutional Symptoms, differing from Papular Eruptions.*

"*Case* 21.—November 13, 1815; a deep ulcer, with hard irregular margin, on the inner membrane of the prepuce. It was of the size of the diameter of a large split pea; and he had bubo in his right groin. The symptoms were of a few days' standing. The sore was healed with a great deal of thickening and hardness. The bubo had suppurated, but the matter was beginning to be absorbed. January 2, 1816; a very irritable sore was again formed, from his having rubbed the cuticle off the cicatrix ten days ago. The whole prepuce was inflamed and swollen. Feb. 7; the sore had again healed. The hardness was like a piece of marble. The bubo had come forward, and had burst and healed. His skin had a dark mottled appearance over every part of his body."

"*Case* 22.—May 23, 1816; a deep foul sore by the edge of the corona glandis, near the frænum, one side of which was destroyed by it. The base and margin were much indurated and thickened, and the discharge was thin and acrid. The sore had been present a fortnight, and came four or five days after suspicious connexion. This sore continued for a long time exceedingly irritable, and was not healed till the 8th of August, and then with considerable hardness. A gland became affected in his left groin a few days after his admission, but was dispersed in about a month. July 6; he observed some spots on his breast and loins, and, in a day or two, his whole body was covered very thick with dark-brown patches, of an irregular form, and a little elevated, larger than the diameters of split peas, giving a mottled appearance to his skin. A few were visible on his forehead, about the roots of his hair, and behind his ears. The sore, at the time this appeared, was not giving much disturbance: he was thin, but his health was good."

"*Case* 23.—On the 24th of May, 1816; a deep sloughy chancre, of six weeks' standing, which had destroyed a portion of the corona glandis. I gave him five grains of the common mercurial pill twice a-day, after he had been in the infirmary for a fortnight: it was continued only four days. His gums were rendered a little turgid. June 25; the sore was healed with much hardness, and an enlarged gland, which had appeared in each groin a few days after his admission, was dispersed. An eruption of a coppery colour, in flat spots, a little elevated, and not regularly circular, about the size of large split peas, had appeared on his back and shoulders."

"*Case* 24.—On the 12th of December, 1815; a sore having the common appearances of chancre. It was healed, with much hardness and thickening, on the 13th of January following, and he was dismissed. April 16; his health has been good, and there has been no appearance of any cutaneous affection, but his hair has come off in large patches, particularly about the back of his head."

"*Case* 25.—On the 24th of January, 1816; a deep, foul, irritable, sore, on the right side of the internal membrane of the prepuce, with hard irregular edges, and considerable thickening. It had been present eight or ten days, and followed a suspicious connexion. It had produced a bubo in his right groin, as large as a hen's egg. The sore was not healed till the middle of March, and then with a very hard cicatrix. The bubo also was troublesome; it suppurated, and burst in different points over diseased glands. March 24. A portion of diseased skin in the groin had sloughed away, and left a clean sore. His right tonsil was enlarged, and he had an eruption of pale coppery spots over his shoulders, neck, and forehead. The spots were of an irregular form, and scarcely any where elevated. He had felt feverish and out of sorts previous to the appearance of these symptoms."

"*Case* 26.—On the 16th of March, 1816; a small, deep, circular, sore, with hard and irregular edges, immediately behind the corona glandis, and two small sores on the outer edge of the prepuce. He had also some degree of phymosis, a purulent discharge from the urethra, and a gland was enlarged to the size of a hazle-nut in his right groin. May 8; the sore behind the corona was beginning to granulate. It had proved very troublesome, several deep sloughs having formed in it at different periods. A pustular eruption had appeared on his body and limbs. The pustules were very small, not much larger than pins' heads, and were on slightly-elevated and dark-red bases. His tonsils were a good deal enlarged, especially the left, and there was some ulceration in the back part of the pharynx. The eruption had commenced two days before; he had some pain in his loins, but no distinct febrile symptoms. May 15; the eruption had extended to his forehead, chin, ears, mouth, and neck. His right ankle swelled at night, and he complained of pain in the upper part of the tendo Achillis. Numerous tubercles could be perceived over all the inner part of his right leg. They were situated under the integuments, felt perfectly moveable, and were about half the size of garden peas. His tonsils, and the absorbent glands about the angles of the jaw, were much enlarged. May 20; the conjunctiva of the left eye was very vascular, and a zone of red vessels surrounded the cornea. He had pain in the globe. The eruption was not altered."

"*Case* 27.—On the 28th of June, 1816; two sores, which he said had been present five or six weeks, and came a few days after a suspicious connexion. They were of a considerable size, and had the common characters of chancre. They were healed with a good deal of hardness on the 26th of July. He had also a bubo in his right groin which subsided. July 29; an eruption had come out over his legs and arms, and slightly over his shoulders. It consisted of dark brown spots, not elevated, but a little scurfy on their surface, varying from the size of a sixpence, to that of the diameter of a large split-pea: the largest were of the faintest colour. He had also inflammation of the periosteum of the right shin-bone, near its middle: it was a little swelled, and very painful to the touch. Both his tonsils were swelled, and slightly ulcerated; he complained of noise in his ears when in bed, but he slept well, and his general health was not affected."

That phagedenic ulcerations, in every part of the body, erosions and exfoliations of the bones, occur independently of syphilis, or any disease arising from venereal contact, is almost too notorious to require to be named. It will be remarked, that, in the third case, the woman affirmed that "for years she had not been in danger of infection." The author refers also to the case of John Whitbread, who solemnly declared that for eleven years he had not been in the way of infection. This man had lost his palate, and a portion of the upper jaw. Both this and the preceding, though not venereal, were cured by mercury. The case of a young gentleman from the army in France is given, who assured Mr. Bell "that he had neither had syphilis or its antidote, yet he has an exfoliation of the alveolar and palatine processes of the upper jaw.

After his cases, the author tauntingly puts a question to Mr Rose,—would *he* also not have given mercury? and an affirmative answer is somewhat confidently anticipated. What Mr. Rose might answer, we cannot divine; but we suspect, had the same question been put to Palmarius, Fallopius, or Ulric de Hutten, with some others, the reply would have been of this sort:—As *you cannot* cure without mercury, use it; but, as *we can*, we shall do without it.

Referring to the accounts delivered by the regimental surgeons of Portugal, France, and Germany, Mr. Bell insultingly asks—is light to break in upon us from these quarters. This is doubtless one of those oversights to which all are liable in the hurry of composition. *Fas est etiam ab hoste doceri;* and, seriously, we think that light on this subject is more likely to emanate from a regimental hospital; and, although it is not impossible that military surgeons may have gone somewhat too far in insinuating that every case is curable by means *generally known*, and without mercury, yet the evidence presented to us is overwhelming and unanswerable. We see nothing, in the light newly thrown on the question, at all to be compared to the flashes of a vivid imagination. It radiates from a mass of facts that requires more for its extinction than has hitherto been brought to play against it. The author is wrong, we think, in ascribing to the situation of soldiers a quality which possesses the power of modifying and of dissipating venereal symptoms. The writer of this review has witnessed numerous examples of facts similar to those adduced by Mr. Rose.

With respect to Mr. Bell's cases, we perhaps may be dull in not being able to discover any one symptom, in all his examples, peculiarly belonging to syphilis; but we have a tolerable recollection of having met with every one of them under circumstances where venereal infection could never have had existence. If we except the third case, the subject of which affirmed, that for years she had not been in danger of infection, and who (being at that moment under the pressure of one of the severest calamities that, in the shape of disease, can visit a human being,) had no possible inducement to deceive her surgeon,—the utmost we can say of any one of them is, that it *may* have been venereal. Does Mr. Bell require to be told, that venereal ulceration possesses nothing by which, in an abstract point of view, it can be recognized? Is there any surgeon of common experience who trusts to the mere appearance of a sore as the means of enabling him to decide on this momentous question? Does he not anxiously ferret out the history of the symptoms in all their stages? Shall we then take the *ipse dixit* of Mr. Bell,—who, in his note-book, contents himself with a mere transcript of the appearances of the sore without the slightest reference to any previous stage of the disease?

Of Mr. Rose's cases we entertain a very different opinion; and although every one of them, we believe, was cured without mercury, many of them seem as pure examples of syphilis as descriptions will make them: in most of them the sores were on the penis, and clearly traced to venereal contact. Upon what ground, then, does Mr. Bell venture to assert, that "there is not any where, in these essays, to be found, the description of the matter at issue, a venereal sore?" They are at least as near the mark as his own.

Mr. Bell has a happy knack of setting up opinions for his adversaries, solely for the gratification of knocking them down. Mr. Rose had told us, for the purpose of showing that the army surgeons possessed peculiar opportunities of examining the progress of venereal symptoms, and the state of the constitution for years after the cure, that he could do what he pleased with his patients without restraint, and had them for a number of years, certain they could not have a change of opinion, nor act contrary to his wishes. This seems innocent enough, and its import obvious; but, Mr. Bell exclaims, "there is no man who has a touch of feeling in him that does not wish the case otherwise, and to what reflections does it lead?" and reminds us, very unnecessarily, "that the regimental surgeon is bound, by every consideration of honour and duty, to act to his men as to his dearest friend," &c. The author might surely have found a more suitable occasion for indulging in his rhetorical display of fine feelings. It is ungenerous to insinuate that these gentlemen have not as much regard for their patients as surgeons in private life; and, in truth, we think their endeavour to supersede the use of a mineral, (which, although specific in its operation in the cure of the venereal disease, yet, in some constitutions, is notoriously destructive,) does equal honour to their heart and head, and must redound eternally to their credit.

It appears, then, from recent papers on this subject, that a vast number of cases of venereal diseases are curable without mercury; and that, in the hands of certain gentlemen, surgeons of intelligence and veracity, every case which came under their care was actually so cured.

It appears also on recurring to the practice of the ancients, that many of our predecessors, of great experience and fame in every age, have been equally successful; and, from an attentive comparison of dates, it is obvious, that they continued long to practice that which they recommended. Under these circumstances, it may become an instructive question,—how to account for the perpetual recurrence of mankind to the use of mercury. The solution, perhaps, is not difficult. The Medico-Chirurgical Reporter has the following remarks on the subject, which, as we cannot improve, we give in their own words.

"He who sets out with a confident assurance that mercury is no longer needful for venereal disease, is likely to abandon his creed on the first failure, and at the same time lose sight of the undoubted fact that many have been, and still may be, cured without it.—Mankind are ever disposed to run into extremes, but on this point, a slight application of the knowledge of history might have prevented an erroneous conclusion. The disease existed and was cured before the use of mercury, as its remedy was known; it cannot then be difficult to conceive, that, in the present day, it may also be occasionally cured by other means: but the distressing consequences, and even the fatality of its attack, in some cases, seemed to indicate that another remedy was wanted. The efficacy of mercury was at length discovered, and practitioners, forgetting how much had before been done without it, fell into the practice of its indiscriminate employment. This mistake is the more easily conceivable, since we find many cases, in which it is unnecessary, are not aggravated by it, especially if appropriate local treatment be employed, which often cures the complaint before it has been given in sufficient quantity to affect the constitution. On the other hand, the protracted disease, the phagædenic ulcerations, the mutilated noses and palate, perhaps more frequently arise from its misapplication than from any other cause.

"Our personal experience leads us to believe that it is impossible, with any known remedy, to cure syphilis, in every instance, without mercury; but we may be deceived. It merits particular notice, that in every age since the disease has been known, practitioners of eminence, and undoubted credibility, have existed, who not only protested against the necessity of using this remedy, but condemned it in the strongest terms, as inconvenient and destructive. Fernelius, a man whose learning, experience, and great reputation cannot be denied, spoke of its being fallacious and cruel. His authority will have more weight when it is recollected that he not only remained firm in this opinion, but his disciple, Palmarius, who had long lived with him in habits of close intimacy, continued in the same sentiments, and employed the same remedies for a period at least of twenty years, when his treatise on the Venereal Disease appeared. Fernelius died about the year 1557. The first edition of the treatise of Palmarius is dated 1578, and he solemnly declares, that he recommends nothing which is not the result of twenty years experience, and that the good effects of his treatment will be manifest to every practitioner who employs it with skill.''

*(To be continued.)*

---

*The Dublin Hospital Reports, and Communications in Medicine and Surgery.* Vol. I. 8vo. Hodges and M'Arthur, Dublin; Cox and Son, London.

*(Continued from vol.* 39, *p.* 499.)

*An Account of the removal of a Tumour which was seated beneath the Angle of the Jaw;* by J. W. Cusack, M.D. Surgeon to Steevens' Hospital and to St. Patrick's Lunatic Asylum.

It was a tumour situated beneath the angle of the jaw; when first seen, about the size of a hazel-nut, moveable and without pain: in the course of six months, it acquired the size of a hen's egg, when it began to grow very rapidly, and produce such inconvenience as to render its removal indispensible.

It now extended from one lobe of the ear to the lower border of the thyroid cartilage, and from beneath the sterno mastoid muscle, which it had protruded outwards beyond the middle line of the throat, between the os hyoides and chin; it was lobulated, and felt cartilaginous; it was free from sensibility, except at the lower part, where the slightest pressure produces acute pain; it was separated from the mouth by the lining membrane to which it was not adherent, and occupied a considerable space within the mouth, on the side of the tongue.

"The patient's voice was inarticulate, his respiration laborious, and deglutition, even of the smallest quantity of solid food, difficult and painful. He swallowed fluids with ease, except ardent spirit, which he had previously been in the habit of using freely, and which, when he attempted to drink, excited so much pain and spasm, that he was obliged to reject it from his mouth instantly.

"The propriety of attempting an operation was a matter of considerable doubt. Although there was no obstacle to the tying of the carotid artery, yet, from the size of the tumour, and the depth to which it had penetrated, great apprehensions were entertained as to the possibility of separating it with safety from the pharynx. It appeared intimately united to the upper part of the larynx; but, except at this point, it did not seem to have formed any close attachments to the surrounding parts, being moveable with as much facility as could be expected from its size and situation. Encouraged by this latter circumstance, urged by the entreaties of the patient, and fully convinced that the rapid progress of the complaint must otherwise terminate his existence, the other surgeons of the hospital considered me justified in attempting the operation.

"Friday, 25th April.—The patient being laid on a table, his head and shoulders slightly elevated, and his face turned to the right side, an incision was made from the lobe of the left ear, along the natural course of the sterno-mastoid muscle, extending to the cricoid cartilage, and dividing the skin, platysma myoides, and superficial fascia. A second incision was made from the centre of the former, parallel to the base of the jaw, and terminating opposite to its symphisis. Dissecting back the flaps, I exposed the anterior surface of the tumour, invested with condensed cellular membrane, and proceeded to detach it, but experienced much difficulty in the attempt, from the great distress in respiration, which was occasioned by the slightest movement of the tumour. Continuing the dissection outwards, beneath the anterior edge of the sterno-mastoid muscle, and opposite to the os hyoides, a large artery was discerned lying on the outer edge of the tumour. This artery proved to be the external carotid pushed from its natural situation. I secured it, (by a single ligature,) which rendered the subsequent part of the operation less embarrassing, by preventing any farther hæmorrhage from the smaller vessels.

"Having laid bare all the anterior surface of the tumour, and succeeded in detaching the posterior connexions of that portion which was covered by the sterno-mastoid muscle, I next attempted to draw it out, that I might separate its attachments on the laryngeal side. This I found to be impracticable, from the difficulty of respiration produced by every attempt to move the tumour. The magnitude and figure also of that portion which was first exposed, so completely concealed the deeper parts, as to preclude me from following them farther with safety. In this dilemma it was proposed to cut away as much of the tumour as had been denuded, and I accordingly removed nearly one-half of its entire substance.

"The situation of the remaining part now became more apparent. On the laryngeal side, the cornu of the os hyoides was intimately connected with it; a portion reached above the jaw, behind the digastric and stylo-hyoid muscles, by which the submaxillary gland was pushed forward on the margin of the lower jaw. The digastric and stylo-hyoid muscles, passing in front of the tumour, were so directly in the way, that it was found necessary to cut them across, to get at that portion which extended into the mouth. Having detached this with the handle of the knife, and drawn it downward with the hook, I cut it away. A third section reduced the tumour to the size of an egg. In this remaining portion, the cornu of the os hyoides was buried, and any attempt to move it caused great pain and difficulty of breathing. While we were considering how we might remove it, an assistant, insinuating his finger behind to ascertain its connexions, brought it away by a sudden effort, and a quarter of an inch of the cornu along with it. An equal portion of the bone, which was softened and rough, was removed with a pair of scissars. On examining the cavity in which the tumour had been situated, we found that the external carotid had been tied immediately beyond where it had given off its branch to the thyroid gland; except the digastric and stylo-hyoid muscles, we could not discover that any other important part had been divided; the membrane connecting the os hyoides to the thyroid cartilage, which appeared most in danger, escaped unhurt."

*On Periostitis, or Inflammation of the Periosteum;* by PHILIP CRAMPTON, M.D. F.R.S. Surgeon-general to the Army and Forces in Ireland.

It is somewhat singular, that although this disease, in its idiopathic form, must have existed from the earliest periods, is remarkable for the intensity of its pain, and the subject of a peculiar treatment known to most surgeons of eminence; yet, as far as we are acquainted, it has never been the subject of a separate essay, or entered into the brain of the nosologist to class it as a distinct malady. The solution of this mystery will probably be, as suggested by Dr. Crampton, that it occurs so generally in connexion with syphilis, that, by one of those blunders common to medical men, it has been considered to belong to that disease alone.

Periostitis, better known by the term Node, is, whether idiopathic or symptomatic of syphilis, more generally met with on the cranium, the tibia, or sternum. When seated in the periosteum of the apices of the fingers, it is known as one of the species of whitlow—*Paronchia Periostei.*

The intolerable pain and constitutional disturbance attendant on the acute form of this disease, arises from a total want of elasticity in the periosteum, which, like tendon and ligament, is insensible, except in a state of inflammation, when its sensibility is excited to the highest degree.

Periostitis exists in a chronic form, and, as may be easily conceived, will be met with in various degrees of intensity between these two extremes of the scale.

"In the paronychia periostei, the pain is excessive, and the constitutional disturbance is proportionably severe. The finger, which is the seat of the disease, and soon afterwards the hand, become swollen and tense almost to bursting; erysipelatous inflammation, attended with œdema, quickly extends over the hand and arm—the fingers become livid, and, if relief be not obtained by a timely operation, the disease terminates in the destruction by sphacelus of one or more of the phalanges. But the mischief does not always end here, as the mortification sometimes extends to the integuments of the hand, and even of the fore arm: deep-seated abscesses are formed among the muscles, and after weeks, or even months, of suffering, the patient may be esteemed fortunate who escapes from a neglected inflammation of the periosteum of a single phalanx, with a stiffened and mutilated hand.

"In the paronychia of the sheath (paronychia tendinei) the suppuration, after having proceeded to a greater or lesser extent along the sheath, finds its way to the surface through a small opening, which soon throws up a painful fungus, perforated in the centre, through which a probe may be readily pressed down to the tendon, but no farther. In the paronychia periostei, on the contrary, the disease, when committed to nature, I believe uniformly produces sphacelus of the soft parts; and the probe being introduced through the slough, passes down at once to the naked bone. I have been led into this short digression upon the subject of paronychia, from a consideration of its vast importance, since there is, perhaps, no disease for the relief of which art can do so much, and nature so little."

The terminations of periosteal inflammation vary: the acute ends in suppuration; the chronic in cartilaginous thickening, absorption of subjacent bone, or in the deposition of an undue quantity of bony matter upon its surface.

Periostitis may occur as a consequence of constitutional derangement, independently of syphilis: as from scrofula, abuse of mercury, or from residence in hot climates. External injury is also a very common cause: the following is an example of the acute stage.

"In the year 1812, J. Sergisson, a working jeweller in Dame-street, about 26 years of age, of a pale complexion, but of rather an athletic form, applied to me for what he termed a disease in the knee. I found him crying out with pain; his face was pale and bedewed with a cold sweat. Upon the upper and flat surface of the tibia there was a diffused tumor of a pale red colour, extending about two inches below the articulation of the knee joint: it was smooth, hard, and exquisitely painful to the touch. The man said, 'he had not slept for twelve nights; that he was exhausted by suffering; and that nothing remained upon his stomach.' His pulse was at 120, small and weak, his tongue was white, but not coated. He told me he had suffered from a similar attack about two years before; that he had been bled with leeches, and blistered, but without relief. A surgeon then applied a caustic plaster, which he said in a short time made a large opening in the skin, and that he soon afterwards obtained relief by the discharge of some matter. The wound remained open for several months, but at length a small scale of bone coming away, it healed in a few days. I immediately made an incision about three inches long down to the bone; the operation seemed to give an unusual degree of pain; the wound bled freely, but I could not discover any traces of purulent matter. On the following morning, he told me that he had passed an excellent night, and was quite free from pain. The wound was dressed superficially, and healed in a short time: in about three weeks he was able to return to his business. In the year 1815 he experienced a similar attack in the same limb, but somewhat lower down upon the tibia. The disease was treated in the same way, and with the same success."

The following illustrates the disease as seated on the pericranium, producing death by extension to the dura mater. While the inflammation was confined to the periosteum of the face, the symptoms were moderate; but, when it extended to the scull and dura mater, the fatal train of symptoms began.

"Master T. aged 14, remarkably tall for his time of life, full, but not muscular, subject to frequent feverish attacks, on the 1st of September, 1816, got a small angry tumor on the right side of the bony arch of the nose. In a day or two it became inflamed and erysipelatous, and occupied all the teguments on the right side of the nose.

"On the 5th feverish symptoms ran so high, although he had used fomentations and purgatives freely, that ℥xii. of blood were taken from the arm. On the 6th, ℔i. was taken with apparent relief. The swelling still continuing to extend, on the 8th, to the right eyelid, the pulse being strong and hard, the bleeding was repeated to ℔i. On the 9th, to Doctors Harty and Archer, in consultation, the disease seemed so far mitigated, that an attention to the state of the bowels alone appeared necessary. In the evening, however, he became comatose, with a slow full pulse, and occasionally delirious: ℔i. of blood was taken, and a blister applied to the neck.

"Such was the history of the case, when I was called to see Master T. on the 10th.

"At the time of my first visit he had a violent convulsive fit; after which he was nearly comatose, his respiration stertorous: antecedent to this, his breathing was represented as having been uniformly good. Pulse about 120, full and throbbing; the nose, right eyelid, and teguments of that side of the forehead, erysipelatous; both eyes closed; the parts were so tender that the slightest touch gave exquisite pain, and roused him from his coma. Some pus had came from the right nostril, and a little had been spit up, mixed with blood.

"The temporal artery was opened by Dr. Harty without loss of time, and ℥20 of blood were taken from the arm; sixteen leeches were also applied to the temples, and the head kept cool with ice. This latter measure appeared to afford considerable mitigation to the pain, but no eventual advantage followed.

"In the evening ℥xx. more of blood were taken from the temporal artery. On the 11th he seemed to suffer much, as he constantly moaned; but was unable, even when roused, to articulate. —A spot over the right eyebrow, to which he with difficulty raised his hand, was the most painful part. On opening the closed palpebræ with my finger, the pupils looked large and immoveable, and a layer of clear coagulable lymph formed a coating over the cornea of both eyes. Leeches, blisters, &c. were all resorted to, but he died, without any convulsive effort, on the morning of the 12th.

"On raising the scalp, the pericranium, covering the os frontis on the right side, appeared thicker than natural, and was in some parts of a dark-red colour; it was completely detached from the subjacent bone, and its *inner* surface was covered with thick purulent matter of a light green colour. On slitting down the membrane as far as the superciliary ridge, a considerable quantity of purulent matter escaped from all sides, and a probe was easily passed backwards into the orbit between the bone and the periosteum, and downwards behind the temporal muscle, as far as the back of the palate.

"On raising the scull cap, the *outer* surface of the dura mater (for an extent corresponding with the limits of the detached pericranium) was found to be separated from the internal table of the scull: it presented a dull and flocculent surface, and was smeared with a greenish puriform mucus. The structure of the *inner* surface of the dura mater was less altered, but it was more generally covered with purulent matter; it was observed to be in considerable quantity on that part of the membrane which invests the anterior part of the right hemisphere, but there was no part of the internal surface of the dura mater which did not exhibit traces of suppuration.

"The pia mater was, perhaps, more vascular than usual; but the arachnoid membrane was perfectly transparent, and no effusion had taken place upon its surface, except upon the upper part of the anterior lobe of the right side; and here the pia mater, for the extent of about two inches, was in a state of suppuration. This diseased part exactly corresponded in situation and extent with a large patch of thickened and inflamed pericranium of a dark-red colour. The brain was perhaps more vascular than natural, and there was about an ounce and a half of water in the ventricles

"The right side of the face was somewhat swollen, and the upper eye-lid was protruded beyond the eyebrow. An obscure fluctuation could be felt beneath the integuments of the upper part of the nose. On cutting down to the bone, a considerable quantity of purulent matter issued from beneath the periosteum, which was found to be in a state of suppuration, and so extensively detached from the subjacent parts that a probe could, with great ease, be passed between that membrane and the bones, either upwards upon the os frontis, backwards along the roof of the orbit into the cranium, or downwards upon the maxillary bone into the mouth."

In the following, the inflammation terminated in a thickening of the pericranium.

"Mary Loudon,* a ribbon weaver, aged 32, was admitted

"* Although this case came under my immediate observation in the hospital, I am indebted to Mr. Hewson, for the daily reports which were taken at the bed-side."

into the Meath Hospital, on the 14th of December, 1812. She complained of intense pain of the head, chiefly affecting the left side—the right arm was wasted and paralytic, and the fingers were contracted—the lower extremities were so feeble that she could not stand erect without support; her speech was faultering; and her articulation indistinct; her form was emaciated; the countenance pale, and expressive of the greatest distress: she seldom ceased to weep, and said that she could get no sleep from the incessant pain of her head. Her pulse was 60, small, weak, and compressible; her stomach rejected all food, and her bowels were torpid. A few hours after she had been received into the hospital she had a strong convulsive fit. There was a tumor about the size of half a walnut over the middle of the left parietal bone; it was soft and elastic at the centre, but firm at the circumference. There was a small opening at the apex, through which a probe could be passed down to the bone. She gave the following account of the disease: About six months before, her husband struck her on the head with the heel of his boot: she was stunned by the blow, but the integuments were not wounded. She soon recovered from the immediate effects of the injury, and continued tolerably well for a month or six weeks; she then, for the first time, observed a swelling, attended with considerable soreness about the part where the blow had been inflicted. The tumor daily became more painful, and she soon began to suffer from severe head-ach, sickness of the stomach, and want of rest; her health began rapidly to decline, and about a month previous to her admission, (and about five months from the period of the injury,) she was seized with an epileptic fit: on recovering, she discovered that she had lost the use of the right arm, that she expressed herself with difficulty, and that she was no longer able to stand without support. Since that period, the fits have frequently recurred, and her health and spirits have rapidly declined.

"On Tuesday the 18th of August, it was determined, in consultation, to lay open the tumor through its whole extent down to the bone, and to remove, by the trephine, a portion of the skull, corresponding in extent with the diseased part of the pericranium; the operation was performed by Mr. Hewson. It was observed, in making the incision, that the pericranium, which formed the bulk of the tumor, exhibited a firm fibrous structure; that it was unusually vascular, possessed a high degree of sensibility, and adhered with such force to the subjacent bone, that it was with difficulty detached in order to make room for the application of the trephine; the bone was rough, in consequence of a considerable portion of the outer table having been removed by absorption, but the firm and vascular adhesion of the pericranium sufficiently evinced that its vitality was unimpaired. When the circular portion of bone was removed, the dura mater bled freely; the membrane seemed to have undergone a change of structure, in all respects similar to that which was observed in the pericranium. The lips of the wound were laid down and dressed simply.

"19th. She had a much better night than she had enjoyed for some weeks; much less pain of the head: complains, however, of chilliness. Pulse 60, soft and small. Vomited once this morning.

"20th. Had no sleep from uneasiness of the head: lips of the wound somewhat swollen. Vomited frequently. Pulse 70. No stool. Ordered cathartic pills, with calomel, and an effervescing mixture.

"21st. No particular change; stomach still irritated; bowels constipated. The purgative medicines were continued.

"22d. At eight o'clock last night had a severe rigor; is at present in a state of insensibility, from which she can with difficulty be roused; breathing laborious, and accompanied with stertor. The countenance in general pale and sunken, but sometimes suddenly suffused with red; pulse 120, small and indistinct; extremities cold; tongue dry, and brown in the middle, bowels still constipated; stomach rejects every thing. Purgatives continued.

"23d. Passed a restless night; was frequently delerious; edges of the wound more swollen, and extremely sensible; bowels have not yielded; pulse 120.

"24th. Had a severe convulsive fit during the night; continual startings; much delirium; an erysipelatous inflammation occupies the parts about the wound, extending behind the right ear down the side of the neck; bowels still costive; the purgatives, in one shape or other, still persisted in; every thing rejected by the stomach.

"25th. The erysipelatous inflammation extended over the face and both the eyes: other symptoms as before.

"26th. The bowels at length yielded in the course of the night; she afterwards obtained some rest, and the countenance this morning is expressive of great relief; she sits up in her bed, and answers distinctly to the questions which are put to her. The erysipelas has decreased, a considerable quantity of healthy pus has flowed from the wound: the thickened dura mater protrudes above the edge of the bone, and seems disposed to slough; the stomach settled, tongue moist, and cleaning at the edges. Pulse 120.

"27th. Slept well last night; erysipelas rapidly declining; the power of the right arm considerably improved; a slough has been detached from the dura mater. From this day forwards every thing proceeded in the most favourable manner. On the first of September she was free from complaint, and the right arm had completely recovered its strength: on the 20th she returned to her occupation."

A case of the sub-acute form is subjoined.

"Mr. B——, aged 23, of a fair complexion, and of rather a delicate frame, consulted me in the month of March 1814, on account of a disease, of which he gave the following history:

"In the spring of 1812, he first complained of a severe pain in the upper part of the tibia of the right leg. The pain usually attacked him after dinner, and continued through the greater part of the night. In the morning he was always relieved, and able to pursue his usual diversions of shooting and hunting; the seat of the pain, however, remained extremely tender to the touch, although the part was neither swelled nor discoloured. He suffered in this way for several weeks; the pain, however, becoming more violent, and the intervals of ease shorter, the leg used to swell at night, particularly about the ankle, and he was no longer able to walk without pain. In this state he applied for advice in Dublin: leeches and blisters were repeatedly applied with temporary advantage, and he was ordered some pills, which he believes were mercurial. The pain, however continued, and his health became materially impaired: he had a regular accession of fever every night, which terminated in a profuse perspiration at about five o'clock in the morning. He now observed that the shape of the limb was somewhat altered; there was no distinct tumor on the tibia, but about two inches below the knee joint, the upper and flat surface of the bone presented a rounded appearance, and the whole of the limb was somewhat œdematous, but the skin was in no part discoloured. In this state he returned to the country, and applied to the late Mr. Pack of Kilkenny, who told him that the disease was ' an inflammation of the periosteum," and advised that an incision should be made through the whole length of the tumor down to the bone. The operation was accordingly performed by Mr. Pack, with the most complete success; the pain was immediately relieved, and, in a few weeks, Mr. B. was restored to perfect health. In 1813, Mr. B. called upon Mr. Astley Cooper, who told him that he had merely to advise a repetition of the operation so judiciously performed by Mr. Pack, in the event of a recurrence of the disease. Early in March 1814, the disease recurred, and Mr. B. called upon me about the latter end of the month: the inflammation had attacked the periosteum about two inches below the seat of the former disease. The pain commenced, as usual, in the afternoon, and continued until about three or four o'clock in the morning. He then fell into a profuse perspiration, which terminated the paroxysm, and he obtained some rest. His appetite, as in the former attack, completely failed him, and he emaciated rapidly. His pulse was never below 80, and his tongue was white, but not coated.*

---

" * Mr. B. noticed a circumstance with respect to the influence of fermented liquors upon this affection, which appears to me to be of considerable importance, as illustrative of the effects of even very small quantities of alkohol, in diseases of an inflammatory nature. He observed at first, that the plan invariably recurred within an hour after dinner, at whatever time he might have taken that meal, and whether the food had been animal or vegetable. Suspecting that this might be connected with the nature of the liquid, rather than the solid matter which he took into his stomach, he left off fermented liquors; on the first day on which he made this change, the pain did not recur until he had been an hour in bed;

"Mr. B. was directed to take three grains of the blue pill, and two of aloes every night, a pint of the compound decoction of sarsaparilla, with two or three drachms of the powder, in the course of the day. As the disease had commenced but a few days, its precise seat was not easily ascertained; taking, however, the part of the tibia which was most sensible to pressure as a centre, the incision was made to extend about an inch above and below this point. The periosteum was found to be about a quarter of an inch thick, its structure was almost cartilaginous, and its sensibility seemed to be much increased. After the pain attendant on the operation had subsided, Mr. B. experienced complete relief, and the wound, which was dressed superficially, healed in a few days. In the course of a week, however, the pain returned with its usual violence, but it had taken up a new seat, having commenced at the lower extremity of the last incision. In a few days a second incision was made, immediately below the former one, and the pain was again completely relieved. The wound on this occasion was dressed to the bottom, and kept open for nearly three weeks; but while it was yet discharging, the pain recurred in the seat of the original disease; it was, however, by no means severe, and Mr. B. was always able to procure a good night's rest by a grain of opium."

Chronic inflammation, though less severe, is not the less formidable as the subjacent bone becomes diseased, which, in the pericranium, is a serious occurrence. The two following cases illustrate the disease in the scull, and on the tibia.

"In the year 1813, a general officer, about forty-seven years old, remarkably well made, and of a constitution naturally vigorous, returned to Ireland from the Peninsula for the recovery of his health. He suffered from a variety of dyspeptic symptoms, such as costiveness, loss of appetite, depression of sprits, and unconquerable languor. His principal complaint, however, was severe head-ach, which chiefly affected him at night. Upon examining the head, I found that the forehead and scalp were covered with flattish tumors, differing in size, from about a quarter of an inch to nearly an inch in diameter. They were slightly painful upon pressure, but seemed to be as hard as bone to the touch. He was directed to take five grains of blue pill every night, and a draught, consisting of infusion of gentian and rhubarb, with a dram of sulphate of magnesia, every morning and noon. His diet was

---

this led him to institute a number of experiments upon the influence of different kinds of fermented liquors, in different quantities: the result was, that the pain could with certainty be excited within an hour, by drinking a glass of any kind of fermented liquor, however weak, and a single dram, by measure, of port wine, diluted with four ounces of water, acted with equal energy as a glass of the undiluted wine."

regulated, and he was ordered to use the tepid salt-water bath three times a week.

"I saw this officer about two months afterwards; his general health was much improved; he had put up a good deal of flesh, and the tumors of the scalp and forehead had completely disappeared; but he still suffered occasionally from head-ach, and sickness of the stomach."

"In the month of May 1816, I was called on to visit a lady from the country, who had, for nearly three years before, laboured under a painful affection of one of her legs. She was twenty-five years of age, of a fair complexion, and apparently delicate habit; but she ascribed her delicacy to repeated attacks of pain, long confinement, want of rest, and the use of medicines to a considerable extent.

"This lady had a swelling on the fore-part of the tibia of the right leg, about two inches above the ancle joint; it was nearly three inches in length, not very prominent, and the skin of its natural appearance. The tumor was soft but elastic, and conveyed a sensation as if a small quantity of fluid was collected under the periosteum. The integuments over the tumor, and for some distance around it, were slightly œdematous. The patient suffered much pain, which was increased by keeping the leg in a depending position, and, as the warmth of the bed always increased her sufferings, she generally passed the night walking about her chamber. In the morning she slept for an hour or two, but was seldom free from pain, except for a short time in the early part of the afternoon.

"Various remedies had been tried by country practitioners of judgment and experience, but without decided benefit. After local treatment had failed, she was put through a full and protracted course of mercury, which had the effect solely of producing considerable debility. During the summer months her pain was by no means so acute as in the cold seasons, and she was induced, in the month of July and August 1814 and 1815, to have recourse to the cold bath; but on the approach of winter her sufferings recurred, and continued to the period at which she placed herself under my care.

"In the various plans of treatment which had been employed for the relief of my patient, local blood-letting was omitted; it was therefore determined, in consultation, that six leeches should be applied to the tumor every evening, and as much blood taken from the part as possible. During the day a compress, frequently wetted with camphorated spirit, was laid on the swelling; the bowels were kept open with small doses of the sulphate of magnesia, and when pain was very severe, an opiate was administered; but this, in general, did not produce a narcotic effect, and was therefore discontinued after the third or fourth exhibition.

"These remedies were persisted in for more than a week; the repeated bleedings weakened our patient considerably, and as the skin became tense, and the relief was very partial, we resolved to make an opening into the tumor. Accordingly an incision of the full length of the swelling was made completely to the bone; the surfaces of the wound bled freely, but no matter was discharged. The integuments were very much thickened, and the parts, close to the bone, had acquired a ligamentary firmness. There was so little retraction of the edges of the cut, that I was obliged to interpose a few dossils of lint to prevent adhesion.

"In the course of a few days the wound suppurated; some relief was obtained, and my patient left town on the 26th of May. In a month after her return to the country, I received a letter from her, to state that the wound had healed, that she felt somewhat better, but that she suffered severe pain occasionally. I have not heard from her since."

---

*Methode Preservative. (Preservative Method.)* Inserted in the New Theory of M. Lenthois, of the Ancient Faculty of Montpelier, respecting Consumption of the Lungs.

[From our Correspondent at Paris.]

This well-written work offers ideas so new on that terrible disorder which it proposes to cure, by means so simple and so easy, that we should be tempted to discredit them, were the wonderful and numerous cures not so well attested.

The practice of Dr. Lenthois goes farther than merely curing those afflicted, (of whom he can cure fifteen in twenty, or three in four,)—he aims at prevention, by removing the tendency to consumption.

This disease does not arise, he says, from any natural defect in the pulmonary tubes, but in a deterioration of the humours, which takes place gradually and silently, that is, imperceptibly. Hence arise those colds, coughs, lymphs, and phlegms, which attack the person at intervals more or less distant, and which finish by attacking the lungs.

The object of this practice is to remove those dangerous humours.

Tartar emetic is the proposed remedy; and the mode of administering is neither difficult, disagreeable, nor expensive:—a grain of tartar emetic is dissolved in eight table-spoonfulls of distilled water; that is mixed with six or eight pints of water, but never more than twelve, nor less than six,—and which is regulated by experience. This is used for drink, either alone or with other drink at meals, or with wine at all seasons and hours, and without any limited time, it being attended with no inconveniency.

Such is the mode of cure, if the disease is begun,—or of prevention, if it is not. Too many persons are interested in its reality, for that not to be soon ascertained beyond any doubt.

